# The Spillover Effects of Spousal Chronic Diseases on Married Couples’ Labour Supply: Evidence from China

**DOI:** 10.3390/ijerph16214214

**Published:** 2019-10-30

**Authors:** Zheng Shen, Xiaodong Zheng, Yiwen Tan

**Affiliations:** 1 School of Economics and Management, Zhejiang A&F University, Hangzhou 311300, China; shenzheng@zafu.edu.cn; 2 School of Economics, Zhejiang Gongshang University, Hangzhou 310018, China; 3 College of Economics and Management, China Agricultural University, Beijing 100083, China; zhengs1988@gmail.com

**Keywords:** chronic disease, spouse, labour supply, spillover

## Abstract

The objective of this study is to examine the spillover effects of chronic diseases experienced by spouses on their wives or husbands’ labour supply. Using data from 2010 and 2012 of the China Family Panel Studies (CFPS), this study employed a difference-in-difference (DD) strategy to investigate the average treatment effect of affected adults on their spouses’ working hours. The results show that, after their spouses were diagnosed with chronic diseases, the average weekly working hours of wives and husbands would be significantly reduced by 3.7–4.2 h and 3.8–4.4 h, respectively. Specially, the average weekly hours of full-time work would be reduced by 2.1–3.3 h for wives and 3.6–3.8 h for husbands. The effect was stronger for those married couples with lower socioeconomic status (SES), such as low-level education, family asset, non-labour income, while the effect was insignificant for high-level SES households. Therefore, as a result of the adverse spillover effects on household labour supply, chronic diseases could cause a greater loss of labour force productivity. Additionally, households in low levels of SES may suffer more losses from reduced labour supply when spousal chronic diseases take place.

## 1. Introduction

With the development of industrialisation and modernisation, China has been experiencing an epidemiological transition in disease patterns from acute infectious diseases to chronic diseases, such as stroke, heart disease and chronic pulmonary disease [1]. The increasing prevalence of chronic diseases could induce a substantial national economic burden by increasing the mortality rate and reducing labour productivity of a large number of working-age individuals. A recent study estimated the “cost-of-illness” of China and found that the five major chronic diseases (cardiovascular disease, cancer, chronic respiratory disease, diabetes, and mental health) would have a total of 23.03 trillion USD economic costs to the country in 2012–2030 [2]. While the majority of the evidence has been found that an individual’s chronic diseases are associated with a significant reduction in their labour market performances [3,4,5,6], few studies concerned whether the chronic diseases experienced by spouses have a similar detrimental effect on their partners’ labour supply.

China is an interesting case for two reasons. First, medical care resources in China are unequally distributed among different institutional settings. Due to low level of professional ability and equipment in primary care institutions, most medical service for patients with chronic conditions comes from hospitals rather than primary healthcare institutions such as community health centres, township health centres, and village clinics [7]. The over-dependence on hospital care leads to long waiting times for patients and their partners. Additionally, although 95% of Chinese population was covered by health insurance in 2011, the reimbursement rate of inpatient care is limited, and the financial risk remains high in the context of universal health insurance coverage [8]. Second, unpaid care work is an important component of Chinese people’s time allocation in labour patterns. According to data from the 2008 China Time Use Survey, the average value of time spent on unpaid care work was 10.6 h per week for males and 27.3 h per week for females, which accounted for 20.2% of total work time for males and 47.1% for females, respectively. Moreover, the estimated monetary value of unpaid care work varies from 25–32% of China’s GDP, from 52–66% of final consumption, and from 63–80% of the gross products of tertiary industry [9].

Since social security systems are not well-functioning and home health services are underdeveloped, households in China are more likely to rely on family members’ informal nursing care to cope with health shocks such as chronic diseases. This coping strategy might be another potential economic burden caused by the indirect cost from spillover effects of spousal chronic diseases, which has been largely neglected in previous studies when implementing a cost-benefit evaluation. In this respect, understanding the association between spousal chronic diseases and their partners’ labour supply, particularly for a typical example of a middle-income country like China, is of great importance.

However, an individual’s labour supply response to chronic diseases from their spouse is theoretically ambiguous. The overall effects can be divided into two aspects: income effect and time effect. On the one hand, the loss of income results from spouses’ labour supply, which is due to health shocks, may lower the unaffected partners’ marginal value of time spent on housework. As such, unaffected husbands or wives would subsequently increase their labour supply to obtain sufficient earnings to compensate for the detrimental financial effects of spousal chronic diseases. On the other hand, if those spouses who have chronic diseases need nursing care, then their partners would tend to spend more time on the assistance of housework and healthcare for the affected spouses. As a result, those unaffected husbands or wives would reduce their working time on the labour market.

Besides, these competing effects may vary by different socioeconomic status (SES). For low SES households, income effect would dominate that lead one to increase labour supply in response to spousal chronic diseases. Because couples with low SES are more likely to be liquidity constrained if they have no access to the social security benefits to smooth consumption. However, people live in low SES households are usually more emotionally vulnerable to be affected by health shocks or undesirable stressful events, for the reason that fewer resources and lacking available coping strategies for them [10]. In this situation, the time effect might be stronger, and thus, an individual would prefer to reduce their hours of work to spend more time on household production for spousal health. Households with high SES, on the other hand, are less likely to change their labour supply decisions when facing a health shock. Because high SES households usually have sufficient resources to afford the expenditure of health insurance, medical care and superior health services, which could contribute to protecting households from the adverse impacts of chronic diseases.

Using data from two waves (2010 and 2012) of the China Family Panel Studies (CFPS), this study employs a difference-in-difference (DD) strategy to investigate the labour supply effects of spouses with chronic diseases on their wives or husbands. Weekly hours of work and weekly hours of full-time employment are the two indicators used to measure the labour supply outcomes in both cases of wives and husbands who are in their working-age (25–64 years old) and employed with a payment. Moreover, we implement several robustness checks and analyse the heterogeneity of the effects by SES to ensure the validity of our results and better understand the spousal spillovers in a family.

Our empirical findings, which are robust to several specification checks, indicate that after spouses diagnosed with chronic diseases, the average weekly working hours of wives and husbands are significantly decreased by 3.7–4.2 h and 3.8–4.4 h, respectively. Furthermore, the average weekly hours of full-time work are reduced by 2.1–3.3 h for wives and 3.6–3.8 h for husbands. The results suggest that the adverse spillover effects on household labour supply would take place when spouses have chronic diseases, which could cause a more significant loss of intensive margin in labour force productivity for national economic growth. Moreover, these detrimental effects are stronger for couples with low SES such as low-level educational attainment, family asset, and non-labour income. However, the results do not appear to be significantly different from zero in terms of high SES households, indicating that high-level SES could alleviate the adverse impacts of chronic diseases on labour supply reduction.

The remainder of the paper is as follows. Section 2 reviews the theory and related literature. Section 3 discusses the data and the empirical method used in this study. Section 4 presents the empirical results, including the main results, differences by SES and robustness checks. Section 5 contains discussion, and a conclusion follows in Section 6.

## 2. Theories and Literature Review

### 2.1. Added Worker Effect (AWE)

The added worker effect (AWE) theory, which was put forward by Lundberg (1985) [11], hypothesises a temporary increase in the labour supply of wives when his husbands are unemployed. In subsequent research, Charles (1999), Coile (2004) and Garcia-Gomez et al. (2013) argued that, when the leisure time is a normal good, an AWE may also happen to wives’ labour supply response to their husbands’ health problem [12,13,14]. The income effect of the losses in earnings, which is induced by the poor health of the primary breadwinner, could motivate females to work longer hours. This effect would be stronger if there is no social security or health insurance to deal with such a health shock from their spouses to mitigate the households’ liquidity constraints.

Using the Health and Retirement Study (HRS), several studies have examined the AWE of spousal health shocks on men and women’s labour market outcomes. Charles (1999) used an instrumental variable approach and found that married women whose husbands are in ill health would significantly increase the annual hours of work, but husbands have a substantial reduction on labour supply in response to their partners’ poor health [12]. Coile (2004) estimated the effects of spousal health shocks on their partners’ labour supply by using six waves of a longitudinal study of the HRS. The results showed that AWE is modest for men’s labour supply and there is no such effect for women [13]. Van Houtven and Coe (2010) constructed a combined sample from the HRS and Social Security Administration (SSA) to examine the impacts of spousal health shocks such as chronic conditions, functional limitations, injury and pain on married individuals’ labour decisions [15]. Their estimates showed that a new functional deficiency experienced by wives would reduce over half an hour of work on husbands’ weekly supply, but the husbands’ health shocks did not significantly affect a married women’s working hours in a typical week.

In essence, the AWE reflects the reallocation of intra-household time resources, which have been examined by Johnson et al. (1975), Parsons (1977) and Berger (1983) [16,17,18]. They showed similar results that, when the husbands’ health deteriorates, wives’ decisions on time allocation between labour market and family would presumably depend on the relative wage rates of husbands and wives and the marginal utility of health care time for the affected husbands. However, the empirical evidence is mixed on the wives’ labour response to their husbands’ health shocks. For example, Johnson et al. (1975) demonstrated that few wives went to work or increased their earnings to compensate for husbands’ disability in the U.S., while Hara (2004) found an increase of 8% in the wife’s labour force participation in response to the husbands’ poor health [19]. In contrast, the results of Hollenbeak et al. (2011) indicated that both wives and husbands of cancer survivors had a significantly higher probability of employment and longer usual weekly hours than their peers [20].

### 2.2. Household Production

In the theory of household production, households are considered to be the producers as well as consumers. Family members combine goods purchased on the market with time inputs to produce commodities, which are the arguments of the utility function while market goods and time are not the desires of households’ own sake but for the inputs of commodities production [21,22]. Based on the household production framework, Grossman (1972) constructed a health production model, in which good health is viewed as the commodity produced by the inputs of consumer’s own time and medical care [23]. Berger (1983) developed a model of the labour supply process in a two-person family and hypothesised that, when spouses had a health problem, the hours of work for unaffected spouses were expected to decrease since the husbands or wives may spend more time on doing housework or nursing care for their affected partners at the expense of labour supply [18]. In another extension of the Grossman health production model, wives and husbands are both regarded as the health producer for each other, given that married couples have a common preference for health production. Meanwhile, they are also the Nash-bargainers, who consider strategically both in the production of their own health and spousal health [24,25,26]. Additionally, the idea of household production was also introduced in a collective labour supply model for a couple’s family, in which individuals would achieve Pareto-efficient allocation of resources for consumption and labour supply by a so-called sharing rule if spousal preferences are egoistic or altruistic [27,28,29].

Empirically, using a cross-sectional sample of individuals aged 35–64, Berger (1983) found that, after controlling for non-labour income, husbands tended to reduce labour force participation likelihood and annual working hours when their wives sufferer from a deterioration in health, which also caused a significant increase of husbands’ time spent on household production [18]. This result was consistent with the theoretical expectation of household production for health investment that the need for nursing care for the spouses, whose health deteriorates, would reduce an individual’s labour supply. In the study of Berger (1983), however, there was no evidence showing that spousal health problem has a negative effect on wives’ labour supply [18]. Instead, married women were more likely to participate in labour market and increase hours of work due to a deterioration in husbands’ health. Similar results were found in the studies of Charles (1999) and Garcia-Gomez et al. (2013), who provided an explanation for this asymmetric effect by gender [12,14]. That is, in a household production model, husbands are assumed to be the primary income earner, while wives are mainly responsible for household work. Thus, spousal health shocks will create either a greater effect of household production by reducing husbands’ working hours or a stronger income effect by increasing wives’ labour supply.

## 3. Data and Methods

### 3.1. Data

The dataset used in this study is the China Family Panel Studies (CFPS), which is a nationally representative and a longitudinal survey conducted by the Institute of Social Science Survey (ISSS) of Peking University starting in 2010. A multi-stage probability proportional to size (PPS) strategy with implicit stratification was performed in the sampling process. The sampling approach comprises three stages: county level as the primary sampling unit (PSU), a community or village for the second-stage sampling unit and the final sampling unit was household [30]. The CFPS survey consists of a rich set of socio-economics questions and information on the levels of child, adult, family, and community. Main variables used in our study are from adult questionnaires, which gather detailed individual information on demographic characteristics, labour supply, disease history, as well as a series of health-related questions such as health status, health care and service, health activities and behaviours. The information is collected and tracked from the face to face or telephone interviews for the age of 18 or above. CFPS was undertaken according to the guidelines laid down in the Declaration of Helsinki and all participants signed an informed consent form. Human participants were approved by the Peking University Biomedical Ethics Committee (IRB00001052-14013-exemption).

To obtain tracked information regarding chronic disease, employment, relevant demographic and socioeconomic characteristics, we construct a two-wave balanced panel data by merging the baseline survey data in 2010 with a follow-up survey in 2012 from the adult and family dataset of CFPS. We restrict our sample to the individuals who are aged 25–64 and work on an active job both in the baseline and the follow-up survey. We also limit our sample to respondents who were married continuously during the period from 2010–2012. The code of spouse in CFPS is used to identify a respondent’s partner in this study. Finally, the sample in this study consists of 848 women and 1608 men in each wave of the survey.

### 3.2. Measures

#### 3.2.1. Spousal Chronic Disease

The CFPS asks respondents to report their chronic disease status, based on the question “have you been diagnosed with any chronic disease over the past six months?” in each wave of the survey. To construct treatment (chronic disease) and control group for spousal disease status in a difference-in-difference approach, spouses who do not have any diagnosed chronic disease in the baseline survey but report a diagnosis of chronic disease at follow-up are selected into a treatment group, which is coded 1. The control group consists of all spouses who do not report any diagnosed chronic disease for the CFPS survey in the baseline and the follow-up, which is coded 0. As a result, the number of cases in the treatment group is 242, in which 76 are women, and 166 are men. Control group has 2214 observations, including 772 women and 1442 men.

#### 3.2.2. Labour Supply

This study measures the labour supply by the weekly hours of work, which are widely used in many studies to examine the health–labour relationship [31,32,33]. In the CFPS data, however, there is no specific question to reflect a respondent’s weekly hours directly. Therefore, we generate the outcome variable by combining two related questions. They are “How many days in a typical month on average did you work for an employed job in the last year?” and “How many hours in a typical day on average did you work for an employed job in the last year?” Using the answers to the above two questions, we first calculate the monthly hours of work through the multiplication of hours of work per day and days of work per month. Next, we obtain weekly hours of work by dividing the monthly hours of work by 4.33 weeks in a month. To test the sensitivity of the association between individual working hours and spousal chronic disease, we also consider an additional outcome measure. That is the weekly hours of full-time work, in which a respondent was employed outside the home and worked at least 35 or more hours per week in the last year.(We divide the sample into females (wives) and males (husbands), therefore, it is no longer needed to control for gender.)

#### 3.2.3. Covariates

In the difference-in-difference (DD) regressions, we further control for individual, spousal, household, and regional characteristics to better reduce estimation bias. First, individual characteristics include age, education, occupation (we divide the sample into females (wives) and males (husbands), therefore, it is no longer needed to control for gender). Among them, age is a continuous variable and three dummy variables (i.e., less than high school, high school, and college or higher) are used to capture the nonlinear effect of education on labour supply. Occupations are also measured by a set of dummy variables, including management or professional (Yes = 1), sales or office (Yes = 1), and farming, construction or others (Yes = 1). Second, spousal health characteristics include whether spouses have been taken cared for by the partner when they are in ill health (Yes = 1), days of hospitalisation in the last year, spousal self-rated health status (poor = 1) and relative health status compared to the health status in the last year (poorer = 1), overweight (Yes = 1) and obesity (Yes = 1) (weight and height are used to calculate the Body Mass Index (BMI), which is based on the equation BMI = weight (kg)/height^2^ (m^2^)). According to the BMI classification for adults, we define an overweight variable as a dummy variable, which equals one if 30 > BMI ≥ 25.0 kg/m^2^, and an obese variable as a dummy variable, which is coded one if BMI ≥ 30.0 kg/m^2^). Third, family characteristics include family assets, non-labour income, family size and the number of children. Among them, family assets and non-labour income are divided into four categories (three dummies) by the quartile scores of their distributions. The number of family members measures the family size and the number of children is the number of children who are aged six or below and live together with parents. Fourth, the differences between rural and urban are captured by a dummy variable that equals one if respondents live in urban areas (urban = 1). Additionally, regional dummies, including east (Yes = 1), northeast (Yes = 1), middle (Yes = 1) and west (Yes = 1) are included in all regressions.

### 3.3. Methods

We seek to estimate the average treatment effect on the treated (ATT), which is a causal impact parameter to compare the difference between an individual’s expected labour supply in the case of spousal chronic diseases and the expected labour supply if their spouse had not been diagnosed with a chronic disease. The ATT can be written as:

(1) $$ATT=E(Y_{1}|T=1)-E(Y_{0}|T=1)$$

where T denotes the treatment status that indicates whether a spouse has a diagnosis of chronic disease; $Y_{1}$ is the labour supply of wives or husbands when their spouses are diagnosed with chronic disease; $Y_{0}$ is the labour supply of those with unaffected spouses. However, the expected labour supply $E(Y_{0}|T=1)$ is not possible to measure since it is usually unobservable in reality. To address this problem, an alternative expected outcome from the control group $E(Y_{0}|T=0)$ is selected to add and subtract on the right-hand side of Equation (1), the results of which are as follows:

(2) $$ATT=[E(Y_{1}|T=1)-E(Y_{0}|T=0)]-[E(Y_{0}|T=1)-E(Y_{0}|T=0)]$$

The selection bias would arise if the expected labour supply of the unaffected spouse groups do not equal to an individual’s labour supply response if their partner had not suffered from chronic diseases, namely, the assumption of $E(Y_{0}|T=0)≅E(Y_{0}|T=1)$ fails to hold [34]. Randomised controlled trial (RCT) design is an ideal method to eliminate this bias. However, it is not feasible in our study because treatment and control groups are not fully randomly assigned in the CFPS. Instead, spousal chronic diseases (treatment) seem to be a naturally occurring or unplanned event that happens to be an exogenous health shock to the labour supply response of their partners. Thus, we use a difference-in-difference (DD) approach, which is regarded as a quasi-natural experimental method and allows for selection bias on both observed factors and unobserved characteristics that are constant over time [35]. In such a setting, any time-invariant confounders (whether observed or unobserved) that might have a potential impact on labour supply should be eliminated by a DD estimator. The DD estimator can be written as:

(3) $$DD=\left(Y_{T,Post}-Y_{T,Pre}\right)-\left(Y_{C,Post}-Y_{C,Pre}\right)$$

where $Y_{T,Post}$ is the labour supply of the treatment group in the second period when the spouse is diagnosed with chronic diseases; $Y_{T,Pre}$ is the outcome of treatment group in the first period when spouse does not suffer from a chronic condition; $Y_{C,Post}$ and $Y_{C,Pre}$ are the outcomes of control group in which spouse has no chronic disease in both two periods. To augment the robustness of the DD estimated results, we use a standard DD linear regression model that allows for several controls of observed characteristics, which is shown as follows:

(4) $$Y_{it}=\beta_{0}+\beta_{1}\left(Treatment_{i}\times Post_{t}\right)+\beta_{2}Treatment_{i}+\beta_{3}Post_{t}+X^{\prime }\theta +\mu_{it}$$

where i refers to the *i*th individual, and t refers to the period *t.* The dependent variable, $Y_{it}$, includes two indicators for labour supply outcomes, including weekly hours of work and weekly hours of full-time work. Among the independent variables, *Treatment* is a binary variable that equals one for treatment group and equals zero for the control group. *Post* is also a binary variable coded one in the post-disease period and coded zero in the period that the spouse does not suffer a chronic condition. The intersection *Treatment* *× Post* is the DD term, and its coefficient $\beta_{1}$ is the DD estimator that captures the ATT of spousal chronic disease on their partners’ labour supply outcome. X is a vector of variables that may be associated with the dependent variables, including individual characteristics such as age and education, spousal health status, socioeconomic characteristics, and regional dummies. Since women and men have very different labour and health characteristics, we estimated separate models for wives and husbands. Furthermore, standard errors in all DD regression specifications are adjusted to cluster with the primary sampling unit at the county level.

The parallel trend assumption, which assumes that the difference between the treatment group and control group remains unchanged over time when there is in absence of treatment, is critical to guarantee the accuracy of the DD estimates [35]. A common approach to meet this assumption is to compare the time trend of outcomes between the treatment group and control group before the treatment (i.e., spousal chronic disease diagnosis in this study) takes place. However, we cannot observe such time trend because the baseline survey of the CFPS was launched in 2010. In order to address this problem, an alternative approach that combines DD regression and weighting on the propensity score is used to check the robustness of our main results. The main idea of this approach is to generate a control group with balanced covariates with the treatment group to allow for a valid comparison by using propensity scores. Given all observed characteristics, the propensity score is the predicted probability of being in the treatment group through a binary logit or probit model. Then, the conditional distribution of covariates could be balanced given a certain propensity score for individuals in the treatment group and control group [36,37]. Specifically, we first estimate the propensity score of spousal chronic disease through a binary logit regression model, where we include a series of explanatory variables related to baseline characteristics that are both associated with the likelihood of disease status and the labour supply among spouses’ wives or husbands. (Explanatory variables to predict propensity score are spouse demographic (gender, age, education and occupation), spouse health characteristics (whether they have been cared for by a partner when suffering ill health, days of hospitalisation, self-assessment of current and previous health status, and whenther thay are overweight or obese), spouse risky health behaviours (smoking and heavy drinking), health insurance, family assets, non-labour income, family size, number of children in household, urban and region dummies). Using the estimated propensity score, we apply a nonparametric kernel matching estimator (Epanechnikov matching with the bandwidth of 0.06 (to test robustness, we also tried other kernel matching estimators including biweight, Normal and Uniform with different bandwidth such as 0.01 and 0.001. The results appear to be robust and similar in all matching procedure)) to generate comparable treatment and control group with similar observable characteristics. In this case, all individuals are observed within the range of common support, and there should be no statistically significant differences between the matched treatment and control group, which suggest that the balancing properties of propensity score are satisfactory after performing a kernel matching. We then assign a weight for each individual based on the estimated propensity score and treatment status [38]:

(5) $$weight_{i}=T_{i}+\left(1-T_{i}\right)\cdot \frac{P_{i}}{1-P_{i}}$$

where $T_{i}$ is the treatment status of the ith person; $P_{i}$ is the propensity score obtained from logit regression. Individuals in the treatment group ($T_{i}=1$) would be assigned the weights that equal to one. For those in the control group ($T_{i}=0$), their weights are equal to the odds of the estimated propensity score. After this propensity score weighting procedure, we run a weighted DD regression with the same covariates in the above DD specification to check the validity of the empirical results.

## 4. Results

### 4.1. Descriptive Statistics

Table 1 presents the descriptive statistics (mean) of the main variables used in this study, which are divided by gender and the treatment status of the baseline and the follow-up survey. Moreover, the P-values of the differences between treatment and control groups are also shown in Table 1. The results show that, in both cases of the baseline and follow-up, the mean age of wives and husbands in the treatment group are significantly larger than those whose partners have never suffered from a chronic disease, while there are no significant differences by treatment status for both cases of wives and husbands in terms of their education levels and occupations.

Among the variables of spousal health, significant differences between the treatment group and control group in spouses’ health status suggest that individuals in spousal chronic disease group have higher rates to have poor health status than the control group. Similar significant differences are found in other health characteristics of spouses at follow-up. For example, compared to control group, those spouses diagnosed with chronic condition have longer average days of hospitalisation (4.75 versus 0.67 for wives and 3.19 versus 0.53 for husbands) and a higher ratio to have worse health status compared to last year (48.7% versus 19.3% for wives and 59.0% versus 24.3% for husbands). Differences of the second quartile and fourth quartile of the family asset between disease and non-disease populations in wives’ samples are significant and larger than those in husbands’ samples, whereas non-labour income quartile, family size and the number of children do not vary significantly by chronic disease status. For those time-invariant observed characteristics such as urban and region dummies, most of their mean differences are statistically insignificant at baseline and the follow-up.

After excluding those observations that fail to meet the requirement of full-time employment (working time below 35 h per week), the remainder of observations for each wave is 2064 (63 and 658 for wives in the treatment and control group; 140 and 1203 for husbands in the treatment and control group).

In the baseline period, differences of labour supply (weekly hours of work and weekly hours of full-time work) between the treatment and control group for wives and husbands are insignificant, which indicate that an individual’s labour supply is initially similar before their spouses suffering from the chronic diseases for both treatment and control group. In contrast, once spouses are diagnosed with chronic disease at follow-up, the average hours of employment per week for wives or husbands in the treatment group appear to be smaller than those in the control group, especially stronger significant differences for husbands. Moreover, there is a noticeable reduction in wives’ and husbands’ labour force supply for the treatment group in response to adverse conditions experienced by partners at follow-up (1.5–3.5 h for wives and 5.5–7.0 h for husbands). These results suggest that a post-treatment effect on wives or husbands’ labour supply might take place when their partners have chronic diseases. Therefore, in the following sections, we implement a DD analysis to examine the change in outcomes before and after treatment groups by comparing the control group.

### 4.2. Main Results

Table 2 reports the results from the standard linear DD estimation of the effects of spousal chronic diseases on weekly hours of work and full-time work. The estimated coefficients of the intersection term *Treatment* *× Post* across all regressions are negative and statistically significant, which indicate that spousal chronic diseases have a spillover and detrimental effect on their partners’ labour supply in both cases of wives and husbands. In the case of wives, after spouses were diagnosed with chronic disease, the time spent on work is reduced by approximately 3.7 h (2.5 h for a full-time job) per week. For husbands, shown in Column (3) and (4) of Table 2, the corresponding effect of spousal chronic diseases on weekly working time is a decrease of 3.8 h and 3.6 h for full-time employment. The impact on husbands is partially a result of the impact of post-treatment (after having a chronic disease), which appear to be negative and significant in the estimation results on variable *Post*. Moreover, none of the point estimates for the treatment status is statistically significant, indicating that little time-invariant differences are found between the treatment and control group across the two periods with controls.

As for control variables, age has a negative and significant association with an individual’s labour supply. The marginal effects of education indicate that working hours or full-time work per week would be significantly reduced when an individual’s levels of education rise. Occupation status is negatively associated with wives’ working hours for both cases of sales or office, and management or professional. We find that nursing care for the spouses being in ill health would lower an individual’s weekly working hours in terms of full-time employment (2.4 h for wives and 1.5 h for husbands, respectively). The point estimates on spouses who are obese show that their partners reduce labour supply and the magnitudes are more significant and considerable among husbands with the results of an average 3.8–5.8 hours’ reduction on weekly working hours. The coefficients on the fourth quartile of family assets are negative and significant, suggesting that people in the wealthiest family tend to have less working time. However, we do not find evidence that family non-labour income has a significant association with individuals’ labour supply. Family size has a significant but modest impact on wives’ labour supply while the number of children in the household does not significantly affect the decisions of wives and husbands on working hours.

### 4.3. Heterogeneity by Socioeconomic Status

The effect of spousal chronic diseases on wives or husbands’ labour supply may vary across characteristics of socioeconomic status. Specifically, we divide our sample into two subsamples, namely less than college and college or higher, and then estimate separately with each subsample. In Panel A of Table 3, the results show that individuals who have an education level less than college are more likely to have less working time (2.9–5.0 h for wives and 4.3–4.6 h for husbands) when their spouses are diagnosed with chronic diseases. For those individuals with college or higher education degree, the coefficients of ATT estimates much smaller and insignificant, implying that wives or husbands do not significantly change their labour supply even if spouses have chronic diseases. Similarly, when we consider the differences between spousal education levels, of which the results are presented in Panel B, the effects of spousal chronic diseases are also negative and significant for those with low-level education (2.7–4.4 h for wives and 4.0–4.3 h for husbands) but insignificant for partners’ education with a college degree or higher.

Next, we explore the heterogeneity across different levels of family asset and non-labour income by dividing the sample into two subgroups. The low groups are defined as those below the sample median of the family asset or non-labour income while high groups are those above the corresponding median. As shown in Panel A of Table 4, there is a stronger negative effect on the low group of family asset, where labour supply reduces by 5.5–7.0 h for wives and 4.7–6.2 h for husbands. In contrast, results on coefficients of ATT for wives and husbands in high group asset are insignificant, which suggest that family asset can mitigate the adverse impacts of health shocks from spousal chronic diseases. In terms of non-labour income, the estimates are very similar to the results of family asset differences. As presented in Panel B of Table 4, when their spouses are diagnosed with chronic diseases, wives with low levels of non-labour income reduce about 5.6 h and 3.4 h for full-time employment, which husbands would decrease by 6.1 and 5.0 h, respectively. However, the labour supply for either wives or husbands with high levels of non-labour income does not have significant responses to their partners’ chronic conditions.

### 4.4. Robustness Checks

We adjust sample, method and variables to check the robustness of this study. First, as the age meet or surpass the retirement age, individuals may reduce their labour supply since they may like to enjoy more leisure time in the company of their spouses if the marginal utility for leisure is higher than that of working on labour market [39,40]. Therefore, we re-estimate the DD models by restricting the analyses sample to workers aged less than the mandatory retirement ages in China (50 years old for women and 60 years old for men). Panel A of Table 5 presents the estimate for non-retired age sample. The results show that wives significantly decrease their working time by an average of 4.1 h per week and 3.3 h per week for full-time work when their spouses are diagnosed chronic diseases, which is slightly higher than the estimates (3.7 and 2.5 h) for the full sample. In the case of husbands, the results are also very similar to those in the main results, which are shown in Table 2.

Second, Panel B in Table 5 displays the results of weighted DD regressions. The weights are the estimated propensity scores obtained from a binary logit model for the probability of spousal chronic diseases at follow-up, which is beneficial to balance the treatment and control group. Effects of spousal chronic diseases on wives and husbands’ hours of work are still negative and significant. Furthermore, the estimated coefficients of ATT are also close to the main results reported in Table 2, suggesting that the DD regression results are robust in this study.

Third, we further check the robustness by including several controls such as risky health behaviours and social security benefits that might be potentially correlated with both the vulnerability to chronic diseases and labour market outcomes. Controls for risky health behaviours include cigarette smoking and heavy alcohol drinking (at least three times a week), which are measured by current and previous status, respectively. The dummy variable for current status of smoking (or heavy drinking) equals one if respondents report they smoked (or drank heavily) last month, and zero otherwise. The dummy variable for former status of smoking (or heavy drinking) equals one if respondents report they did not smoke (or drink heavily) last month but have ever smoked (or drunk heavily) in their past lifetime, and zero otherwise. Controls for social security benefits include family transfer payments and whether the spouse has health insurance. Compared to the estimates on previous full specifications, the results of coefficients in Panel C of Table 5 are still negative and significant, and quite similar to other estimates. Overall, our results show that there is little difference between the estimates of robustness checks and the estimates from the main regressions listed in Table 2.

## 5. Discussion

Theoretically, the response of a partner’s own capacity for labour in light of spousal chronic diseases is ambiguous and depends on which of the competing effects (income effect and time effect) dominate the association of this pattern. If the partners’ marginal value for household production, such as nursing care, needed by the affected patient is higher than economic compensation for the loss of income, they would spend more time with spouses and thus reduce market hours. Otherwise, they would increase labour supply if their value on household production appears to be lower than the working wages. In our empirical analyses, the spillover effects of spousal chronic diseases are found to be negatively associated with their partners’ working hours. Our results are robust to different specifications, which suggest that, in the context of China, the time effect is larger than the income effect and spousal chronic diseases could cause a more significant economic burden on the economic growth of the country.

Similar results are also shown in previous health–labour research of Charles (1999), Van Houtven and Coe (2010) and Garcia-Gomez et al. (2013) [12,14,15], where men work significantly less in response to the health shocks from their spouses, while there is little change in terms of women’s labour supply. However, a recent study by Hollenbeak et al. (2011) found that neither wives nor husbands of cancer survivors reduce their weekly hours. Instead, they tend to work more hours per week than their peers whose spouses are without any cancer diagnosis, which is at odds with our findings [20]. There are several possible reasons for the differences. First, unlike the study of Hollenbeak et al. (2011) focusing on the effect of cancer survivors, we only focus on an aggregate measure that contains multiple types of chronic conditions. Although cancer is one of these conditions, the sample size for cancer survivors is too small to have the dominant effect in our study. In addition, compared to the effect of cancer, other types of chronic conditions may cause less or moderate economic costs through the loss of labour productivity or medical expenditure.

Furthermore, up to the end of 2011, universal health insurance coverage has been successfully achieved in China, where more than 95% of populations were insured [7]. As the expansion of insurance coverage, more people in ill health can get access to health services they need by removing financial barriers to healthcare. Therefore, a partner would have less incentive to engage in labour work to compensate for money spending on their chronic-diseased spouses’ healthcare expenditure.

It is essential to consider a few potential limitations in the present study. One limitation is that, due to the data constraints, we only have two waves of CFPS data and we are not able to identify the parallel trend on the labour supply of the treatment and control group, which is a crucial assumption for DD analyses. If this specific assumption is violated, the estimates of the spousal spillover effects could be biased. Although we used weighted DD strategy to balance the treatment group and control group to meet the parallel trend assumption as far as possible, and the results are quite robust, researchers still should be cautious about making generalisations from our findings. Therefore, for future studies, using data spanning a more extended period on following CFPS or other feasible panel studies would be better to perform a more rigorous DD study on the effects of spousal chronic diseases on married couples’ labour supply.

Second, the objective measures of spousal chronic diseases are not available in the CFPS data. We therefore used relevant self-reported indicators, which might have caused some attenuation biases in our estimated coefficients due to the response error in self-reported chronic conditions [41]. However, this may not be problematic in our study, since we control for a series of spousal health-related characteristics and also check the robustness of estimated results with risky health behaviours such as smoking and heavy drinking.

Finally, this study does not examine the heterogeneity among different types of spousal chronic diseases affecting their partners’ labour supply. The reason is that when using a single dummy variable for a specific chronic disease such as cardiovascular diseases, cancer, chronic respiratory diseases or diabetes, the number of observations for each specific situation turns out to be so small that the sample size is not large enough for our regression analyses.

## 6. Conclusions

Using data from the China Family Panel Studies (CFPS), this study investigates the spillover effects of spousal chronic diseases on their wives or husbands’ labour supply, which are measured by weekly hours of work and full-time work, respectively. The difference-in-difference (DD) method is employed to estimate the average treatment effect of affected spouses on the treated labour supply by constructing the treatment (chronic disease) and the control group. We also examine the heterogeneity by socioeconomic status, including education level, family asset, and non-labour income. Furthermore, robustness checks for the estimated results are implemented in several ways, such as propensity score weighted DD regression, restricting the sample to the age before mandatory retirement age, and adjusting controls.

The results, which are robust in different ways we have checked, show that individuals will reduce their own working hours in response to the spousal chronic diseases. The results indicate that spousal chronic diseases will cause a greater loss of intensive margin in labour productivity for national economic growth due to the adverse spillover effects on household labour supply. We also find heterogeneity among different SES characteristics. Specifically, wives or husbands in the treatment group from households with lower SES (i.e., low-level education, family asset, and non-labour income) work fewer hours per week after their spouses are diagnosed with chronic illness, but wives or husbands with high SES have no significant change in working hours. This result implies that households in a low level of SES will suffer more losses of labour productivity from the affected spouses while a higher SES could mitigate the detrimental impacts on labour supply reduction.

## Figures and Tables

**Table 1:** The mean of main variables for wives and husbands by treatment status at baseline and follow-up.

Variables	Wives						Husbands					
	Baseline			Follow-up			Baseline			Follow-up		
	Treatment	Control	*p*-Value	Treatment	Control	*p*-Value	Treatment	Control	P-value	Treatment	Control	*p*-Value
Age	40.737	38.425	0.013	42.711	40.416	0.014	45.265	42.150	0.000	47.229	44.125	0.000
Education												
Less than high school	0.539	0.534	0.923	0.539	0.534	0.923	0.572	0.601	0.482	0.572	0.601	0.482
High school	0.197	0.218	0.683	0.197	0.218	0.683	0.259	0.221	0.260	0.259	0.221	0.260
College or higher	0.263	0.249	0.782	0.263	0.249	0.782	0.169	0.179	0.744	0.169	0.179	0.744
Occupations												
Farming, construction	0.237	0.335	0.080	0.276	0.333	0.317	0.578	0.533	0.264	0.548	0.533	0.716
Sales or office	0.487	0.399	0.137	0.474	0.398	0.198	0.235	0.229	0.860	0.265	0.255	0.768
Management or professional	0.250	0.236	0.781	0.250	0.258	0.883	0.157	0.200	0.185	0.169	0.198	0.362
Spousal health												
Cared by partner	0.921	0.917	0.905	0.829	0.828	0.978	0.843	0.822	0.489	0.705	0.737	0.372
Days of hospitalisation	0.987	0.482	0.309	4.750	0.674	0.000	1.572	0.563	0.002	3.193	0.533	0.000
Poor status	0.211	0.043	0.000	0.342	0.058	0.000	0.169	0.080	0.000	0.325	0.107	0.000
Worse than last year	0.237	0.170	0.143	0.487	0.193	0.000	0.295	0.221	0.030	0.590	0.243	0.000
Overweight	0.355	0.293	0.256	0.421	0.316	0.062	0.241	0.184	0.075	0.217	0.193	0.459
Obese	0.039	0.039	0.979	0.039	0.038	0.934	0.006	0.017	0.295	0.024	0.025	0.946
Asset quartile												
1st	0.158	0.214	0.254	0.184	0.198	0.770	0.283	0.270	0.728	0.283	0.278	0.891
2nd	0.118	0.240	0.016	0.118	0.246	0.012	0.235	0.264	0.416	0.193	0.265	0.044
3rd	0.289	0.278	0.839	0.276	0.282	0.911	0.193	0.239	0.181	0.223	0.234	0.740
4th	0.434	0.268	0.002	0.421	0.273	0.007	0.289	0.226	0.069	0.301	0.223	0.023
Non-labour income quartile												
1st	0.250	0.302	0.346	0.289	0.337	0.404	0.247	0.230	0.628	0.253	0.248	0.878
2nd	0.237	0.251	0.782	0.158	0.199	0.384	0.313	0.235	0.026	0.181	0.244	0.069
3rd	0.289	0.214	0.129	0.276	0.203	0.137	0.229	0.270	0.259	0.265	0.272	0.852
4th	0.224	0.233	0.852	0.276	0.260	0.763	0.211	0.265	0.132	0.301	0.236	0.066
Family size	3.921	3.951	0.871	3.776	3.949	0.346	3.976	4.051	0.535	3.970	4.078	0.397
Children aged six or below	0.224	0.258	0.553	0.211	0.188	0.654	0.145	0.285	0.001	0.139	0.233	0.016
Urban	0.789	0.750	0.447	0.789	0.750	0.447	0.675	0.641	0.397	0.675	0.641	0.397
Region												
East	0.566	0.478	0.144	0.566	0.478	0.144	0.446	0.451	0.889	0.446	0.451	0.889
Northeast	0.145	0.170	0.579	0.145	0.170	0.579	0.145	0.161	0.587	0.145	0.161	0.587
Middle	0.237	0.222	0.759	0.237	0.222	0.759	0.235	0.239	0.902	0.235	0.239	0.902
West	0.053	0.131	0.048	0.053	0.131	0.048	0.175	0.148	0.371	0.175	0.148	0.371
Weekly hours of work	48.469	49.739	0.497	44.851	48.518	0.083	52.849	52.143	0.628	45.762	49.474	0.018
Weekly hours of full-time work	50.390	52.471	0.257	48.875	52.122	0.106	54.602	55.072	0.743	49.129	53.628	0.003
Observations	76	772		76	772		166	1442		166	1442	

Note: *p*-Value corresponds to the two-sided test of the hypothesis that treatment and control groups have the same mean.

**Table 2:** Effects of spousal chronic disease on wives and husbands’ labour supply.

Variables	Wives			Husbands	
	Weekly Hours of Work	Weekly Hours of Full-Time Work		Weekly Hours of Work	Weekly Hours of Full-Time Work
Treatment × Post	−3.769 ***	−2.535 **		−3.830 ***	−3.643 ***
	(1.282)	(1.148)		(1.456)	(1.396)
Treatment	−0.058	−0.795		1.084	−0.163
	(1.511)	(1.409)		(1.372)	(1.400)
Post	−0.623	−0.125		−2.618 ***	−1.530 ***
	(0.605)	(0.560)		(0.579)	(0.560)
Age	−0.234 ***	−0.066		−0.165 ***	−0.064
	(0.086)	(0.074)		(0.052)	(0.049)
Education: Less than high school					
High school	−3.441 ***	−3.153 **		−3.622 ***	−3.560 ***
	(1.236)	(1.258)		(1.089)	(0.981)
College or higher	−7.004 ***	−7.904 ***		−7.495 ***	−9.175 ***
	(1.242)	(1.160)		(1.247)	(1.311)
Occupations: Farming, construction or others					
Sales or office	−3.873 ***	−1.832		0.801	1.083
	(1.255)	(1.290)		(1.034)	(1.126)
Management or professional	−6.804 ***	−4.039 ***		−3.092 ***	−1.736
	(1.523)	(1.397)		(1.135)	(1.105)
Spousal health					
Cared by partner	−1.519	−2.377 **		−2.110 **	−1.471 **
	(1.436)	(1.189)		(0.844)	(0.689)
Days of hospitalisation	0.046	0.029		0.021	0.010
	(0.048)	(0.053)		(0.052)	(0.054)
Poor status	2.612	3.249		−0.120	1.041
	(2.550)	(2.118)		(1.409)	(1.363)
Worse than last year	2.127 *	1.414		−0.536	−0.927
	(1.156)	(1.129)		(0.681)	(0.662)
Overweight	−1.423	−2.196 ***		1.042	1.168
	(0.863)	(0.758)		(0.875)	(0.895)
Obese	−0.543	−1.691		−5.793 ***	−3.867 **
	(2.368)	(1.590)		(2.218)	(1.825)
Asset: 1st quartile					
2nd quartile	−0.329	−0.483		0.957	0.185
	(1.539)	(1.411)		(1.212)	(1.146)
3rd quartile	−1.337	−2.374 **		−0.936	−1.861 *
	(1.410)	(1.162)		(1.047)	(1.063)
4th quartile	−3.928 **	−4.839 ***		−2.167*	−2.748 **
	(1.621)	(1.362)		(1.188)	(1.276)
Non-labour income: 1st quartile					
2nd quartile	1.510	1.049		0.717	0.651
	(1.029)	(1.041)		(0.977)	(1.034)
3rd quartile	0.727	1.809*		0.590	0.593
	(1.185)	(1.044)		(1.104)	(1.111)
4th quartile	0.997	0.728		−1.748*	−0.661
	(1.148)	(1.007)		(0.983)	(1.043)
Family size	−0.800 **	−0.488 **		0.324	0.548*
	(0.332)	(0.228)		(0.311)	(0.311)
Children aged six or below	1.488	1.750		−0.896	−0.338
	(1.351)	(1.306)		(0.716)	(0.711)
Urban	−1.871	−3.679 ***		−0.333	−1.528
	(1.414)	(1.335)		(0.966)	(1.003)
Region: East					
Northeast	2.251	2.130		6.538 ***	5.719 ***
	(1.770)	(1.527)		(1.374)	(1.368)
Mid	2.019 *	1.432		1.995 *	2.640 ***
	(1.187)	(1.002)		(1.030)	(0.959)
West	−0.231	3.006		1.283	2.884 **
	(2.436)	(2.124)		(1.440)	(1.238)
Constant	69.825 ***	66.191 ***		61.446 ***	59.212 ***
	(5.523)	(4.799)		(3.154)	(2.980)
Observations	1696	1442		3216	2686
R-squared	0.139	0.197		0.090	0.120

Note: Robust standard errors with a cluster at the county level are presented in parenthesis. ***, ** and * indicates significance level at 1%, 5% and 10%, respectively.

**Table 3:** Different effects by individuals’ own education and their spousal education.

Variables	Wives		Husbands		Wives		Husbands	
	Weekly Hours of Work	Weekly Hours of Full-Time Work	Weekly Hours of Work	Weekly Hours of Full-Time Work	Weekly Hours of Work	Weekly Hours of Full-Time Work	Weekly Hours of Work	Weekly Hours of Full-Time Work
	Lower than College				College Degree or Higher			
*Panel A: Education*								
Treatment × Post	−5.000 ***	−2.913 *	−4.624 ***	−4.359 ***	0.845	0.007	−0.439	−1.036
	(1.697)	(1.503)	(1.628)	(1.584)	(1.745)	(1.787)	(2.558)	(2.429)
Treatment	−0.639	−1.767	0.689	−0.615	0.726	−0.443	1.533	0.162
	(1.838)	(1.828)	(1.628)	(1.717)	(2.463)	(2.496)	(1.643)	(1.508)
Post	−0.426	0.068	−2.500 ***	−1.539 **	−1.585 *	−0.981	−3.403 ***	−1.707 **
	(0.764)	(0.746)	(0.659)	(0.646)	(0.803)	(0.665)	(0.964)	(0.767)
Observations	1272	1076	2644	2188	424	366	572	498
R-squared	0.100	0.121	0.062	0.064	0.093	0.103	0.116	0.112
*Panel B: Spousal education*								
Treatment × Post	−4.442 ***	−2.700 *	−4.301 ***	−4.090 ***	−1.049	−2.040	−0.776	−1.708
	(1.646)	(1.496)	(1.560)	(1.507)	(2.200)	(2.250)	(3.041)	(2.984)
Treatment	−0.760	−1.747	0.880	−0.456	1.869	1.013	−0.610	−1.209
	(1.753)	(1.755)	(1.525)	(1.612)	(3.013)	(2.942)	(2.739)	(2.684)
Post	−0.973	−0.288	−2.850 ***	−1.756 ***	0.259	−0.029	−1.568	−0.722
	(0.723)	(0.710)	(0.633)	(0.601)	(0.886)	(0.662)	(1.410)	(1.556)
Observations	1294	1098	2810	2324	402	344	406	362
R-squared	0.098	0.128	0.073	0.079	0.140	0.159	0.126	0.124

Note: All regressions include a constant and control for individual age and occupations, spouse health characteristics (whether have been cared by partner when got ill health, days of hospitalisation, self-assessment of current and previous health status, overweight and obese), family asset, non-labour income, family size, number of children in household, urban and region dummies. Robust standard errors with a cluster at the county level are presented in parenthesis. ***, ** and * indicates significance level at 1%, 5% and 10%, respectively.

**Table 4:** Different effects by family asset and non-labour income.

Variables	Wives		Husbands		Wives		Husbands	
	Weekly Hours of Work	Weekly Hours of Full-Time Work	Weekly Hours of Work	Weekly Hours of Full-Time Work	Weekly Hours of Work	Weekly Hours of Full-Time Work	Weekly Hours of Work	Weekly Hours of Full-Time Work
	Low Group		Low Group		High Group		High Group	
*Panel A: family asset*								
Treatment × Post	−7.021 **	−5.490 *	−6.202 ***	−4.775 **	−2.125	−0.996	−2.023	−2.628
	(3.236)	(3.008)	(2.209)	(2.153)	(1.374)	(1.421)	(1.808)	(1.718)
Treatment	−0.215	−0.787	3.047	1.821	−0.430	−1.459	−0.190	−1.494
	(3.178)	(2.970)	(2.224)	(2.249)	(1.623)	(1.587)	(1.340)	(1.399)
Post	0.301	0.815	−2.169 **	−1.741 **	−1.633 **	−1.105	−3.016 ***	−1.274
	(1.156)	(1.061)	(0.854)	(0.802)	(0.755)	(0.675)	(0.863)	(0.842)
Observations	737	628	1719	1425	959	814	1497	1261
R-squared	0.129	0.173	0.076	0.092	0.140	0.174	0.077	0.111
*Panel B: Non-labour income*								
Treatment × Post	−5.653 **	−3.465 *	−6.105 ***	−5.037 **	−2.255	−1.499	−1.321	−1.440
	(2.275)	(2.013)	(2.253)	(2.283)	(2.323)	(2.162)	(2.283)	(1.882)
Treatment	1.234	−0.180	3.061	1.085	−1.616	−2.331	−1.739	−2.505
	(1.931)	(1.841)	(1.936)	(2.222)	(2.340)	(2.352)	(2.144)	(1.871)
Post	−1.268	−0.526	−2.534 **	−2.291 **	−0.219	−0.019	−2.681 ***	−0.837
	(0.973)	(0.903)	(1.024)	(0.938)	(0.893)	(0.854)	(0.895)	(0.873)
Observations	912	778	1545	1325	784	664	1671	1361
R-squared	0.140	0.199	0.087	0.115	0.148	0.190	0.091	0.133

Note: Low group for family asset and non-labour income are defined as below the median; high group for family asset and non-labour income are above the median. All regressions include a constant and control for individual age, education and occupations, spouse health characteristics (whether have been cared for by a partner when they suffered ill health, days of hospitalisation, self-assessment of current and previous health status, and whether they are overweight or obese), family size, family asset (regressions in Panel A are omitted), non-labour income (regressions in Panel B are omitted), number of children in household, urban and region dummies. Robust standard errors with a cluster at the county level are presented in parenthesis. ***, ** and * indicates significance level at 1%, 5% and 10%, respectively.

**Table 5:** Robustness checks.

Variables	Wives		Husbands	
	Weekly Hours of Work	Weekly Hours of Full-Time Work	Weekly Hours of Work	Weekly Hours of Full-Time Work
*Panel A: Restricting the sample*				
Treatment × Post	−4.087 **	−3.297 **	−4.085 ***	−3.808 ***
	(1.594)	(1.429)	(1.475)	(1.346)
Treatment	−0.038	−0.587	1.908	0.429
	(1.582)	(1.544)	(1.391)	(1.386)
Post	−0.673	−0.166	−2.612 ***	−1.424 **
	(0.667)	(0.608)	(0.578)	(0.561)
Observations	1529	1311	3092	2596
R-squared	0.139	0.195	0.094	0.124
*Panel B: Weighted difference-in-difference*				
Treatment × Post	−4.229 ***	−2.135 **	−4.401 ***	−3.798 ***
	(1.470)	(1.038)	(1.442)	(1.339)
Treatment	0.238	0.105	1.502	0.185
	(1.490)	(1.244)	(1.373)	(1.366)
Post	0.267	0.404	−1.891 **	−0.948
	(1.060)	(0.586)	(0.760)	(0.720)
Observations	1696	1442	3216	2686
R-squared	0.203	0.289	0.131	0.169
*Panel C: Additional controls*				
Treatment × Post	−3.726 ***	−2.367 **	−3.860 ***	−3.756 ***
	(1.297)	(1.181)	(1.461)	(1.408)
Treatment	−0.144	−0.881	1.139	−0.071
	(1.531)	(1.423)	(1.374)	(1.411)
Post	−0.728	−0.026	−2.351 ***	−1.129 *
	(0.635)	(0.613)	(0.644)	(0.637)
Observations	1696	1442	3216	2686
R-squared	0.144	0.203	0.090	0.121

Note: All DD regressions include a constant and control for individual age, education and occupations, spouse health characteristics (whether have been cared for by a partner when they suffered ill health, days of hospitalisation, self-assessment for current and previous health status, and whether they are overweight or obese), family asset, non-labour income, family size, number of children in household, urban and region dummies. DD regressions in Panel B are weighted by the estimated propensity score. Additional controls reported in Panel C include spouses’ current and former cigarette smoking status, spouses’ current and former heavy drinking alcohol (at least three times a week) status, family transfer payments, and whether the spouse has health insurance. Robust standard errors with a cluster at the county level are presented in parenthesis. ***, ** and * indicates significance level at 1%, 5% and 10%, respectively.
